# Sexually transmitted infections and HIV in the era of antiretroviral treatment and prevention: the biologic basis for epidemiologic synergy

**DOI:** 10.1002/jia2.25355

**Published:** 2019-08-30

**Authors:** Myron S Cohen, Olivia D Council, Jane S Chen

**Affiliations:** ^1^ UNC School of Medicine Institute for Global Health & Infectious Diseases Chapel Hill NC USA; ^2^ Department of Microbiology and Immunology UNC Chapel Hill NC USA; ^3^ Department of Epidemiology Gillings School of Global Public Health UNC Chapel Hill NC USA

**Keywords:** STI, STD, HIV, ART, PrEP, shedding, acquisition, transmission

## Abstract

**Introduction:**

HIV is a unique sexually transmitted infection (STI) that is greatly affected by other concomitant “classical” bacterial and viral STIs that cause genital ulcers and/or mucosal inflammation. STIs also serve as a marker for risky sexual behaviours. STIs increase infectiousness of people living with HIV by increasing the viral concentration in the genital tract, and by increasing the potential for HIV acquisition in people at risk for HIV. In addition, some STIs can increase blood HIV concentration and promote progression of disease. This review is designed to investigate the complex relationship between HIV and classical STIs.

**Discussion:**

Treatment of STIs with appropriate antibiotics reduces HIV in blood, semen and female genital secretions. However, community‐based trials could not reliably reduce the spread of HIV by mass treatment of STIs. Introduction of antiretroviral agents for the treatment and prevention of HIV has led to renewed interest in the complex relationship between STIs and HIV. Antiretroviral treatment (ART) reduces the infectiousness of HIV and virtually eliminates the transmission of HIV in spite of concomitant or acquired STIs. However, while ART interrupts HIV transmission, it does not stop intermittent shedding of HIV in genital secretions. Such shedding of HIV is increased by STIs, although the viral copies are not likely replication competent or infectious. Pre‐exposure prophylaxis (PrEP) of HIV with the combination of tenofovir disoproxil fumarate and emtricitabine (TDF/FTC) prevents HIV acquisition in spite of concomitant STIs.

**Conclusions:**

STIs remain pandemic, and the availability of ART may have led to an increase in STIs, as fear of HIV has diminished. Classical STIs present a huge worldwide health burden that cannot be separated from HIV, and they deserve far more attention than they currently receive.

## 1 Introduction

HIV is primarily a sexually transmitted infection (STI) 1. A single sexual encounter between an HIV‐positive partner and an HIV‐negative partner (a serodifferent/serdiscordant couple) has a low probability of HIV transmission 2, 3, 4, 5. When transmission occurs, a single viral variant (the transmitted founder virus) is detected 80% of the time, and usually only a maximum of two or three viral variants are transmitted 6, 7. The transmission of HIV is generally relatively inefficient, and predicted to require hundreds of exposures in the case of penile‐vaginal intercourse 2, 3 and dozens of exposures for penile‐rectal exposure 4, 5.

Such inefficient transmission has made it difficult to understand the magnitude of the HIV pandemic. In part, this can be explained by transmission from HIV‐positive people who do not know their status over many years of asymptomatic infection. HIV transmission reported in stable discordant couples before availability of antiretroviral treatment (ART) was as high as 8.2 to 12.0 per 100 person‐years 8, 9. In addition, several factors could amplify HIV transmission 10. Among the most important amplifying factors are the “classical STIs,” loosely defined bacterial and viral infections that cause genital ulcers and genital mucosal inflammation. Classical STIs are among the most common acute conditions worldwide and have increased in recent years; the World Health Organization (WHO) estimates more than one million incident curable STIs worldwide each day 11. The purpose of this article is to examine the relationship between the classical STIs and HIV with an emphasis on changes in the nature of this interaction since the availability of antiretroviral agents for the treatment and prevention of infection.

## 2 Discussion

### 2.1 STIs in people with HIV

The connection between classical STIs (that cause mucosal inflammation or ulcers) and HIV surfaced early in the epidemic 12 and was first referred to as “epidemiologic synergy” by Wasserheit 13. Subsequent studies have paid considerable attention to biologic mechanisms to explain how STIs promote HIV transmission 12, 13, 14, 15, 16. Such research studies suggested two important roles for STIs: increased infectiousness of the HIV‐positive person and increased susceptibility of the HIV‐negative person 17. Increased infectiousness appears to reflect increases in HIV concentration in genital secretions and changes in viral phenotype of HIV variants that favour transmission.

### 2.2 HIV in genital secretions

Cohen *et al*. studied HIV in semen of men with concomitant gonorrhoea 18 and trichomonas 19 and noted a significant increase in viral concentration relative to a control group without urethritis; the increase in HIV in semen was reduced by appropriate antibiotic treatment, albeit only after several weeks. Shedding of HIV in semen also increases with CMV and perhaps other herpes virus co‐infections 20. Similar increases in the detection of HIV in female genital secretions in the presence of STIs and inflammation have been reported 14, 21, 22, although such findings have not always been consistent 23. Cohen *et al*. reported increased HIV in female genital secretions with bacterial vaginosis, with significantly increased risk of HIV transmission to sexual partners 24.

Indeed, higher concentrations of HIV in blood 9 and genital secretions 25 increase the probability of HIV transmission. The increases in concentration of HIV detected in genital secretions with STIs could reflect increased replication of the virus, an influx in the number of HIV‐infected cells into the genital mucosa, and/or increased exudation of contaminated blood and fluids in ulcerated or denuded mucosal epithelium 17.

### 2.3 HIV‐1 compartmentalization in the genital tract

Over the course of untreated infection, a diverse quasispecies emerges within an individual 26. The emergence of multiple viral variants can be attributed in part to the error‐prone replication of HIV‐1 26, 27, as well as selective pressure from the host's immune system 28, 29. However, as noted above, most new HIV‐1 infections are initiated with a single, or at most a few, viral variants 6, 7 emphasizing the idea that there is a “bottleneck” or “sieve” at the point and time of mucosal transmission 27.

Regional (compartmental) differences in viral diversity can be observed when virus that has been sequestered in an anatomic region undergoes replication independently from virus circulating in the blood. Over time, this independent replication can result in the formation of genetically distinct, compartmentalized viral populations. This phenomenon has been extensively studied in the central nervous system 30, 31 and the male 32, 33, 34 and female 35, 36, 37 genital tracts.

For example, early on Ping and colleagues 34 utilized a heteroduplex tracking assay to analyze the HIV‐1 variants present in the blood plasma and seminal plasma of men from Malawi with and without symptomatic urethritis. The authors hypothesized that in the presence of an inflammatory STI, T‐cell trafficking to the male genital tract would be increased, thus bringing potentially infected cells from the blood into the genital tract and causing the viral populations from the two compartments to mix. In the absence of inflammation, there is less exchange of cells between the male genital tract and the periphery, which would support the formation of genetically distinct compartmentalized viral populations. Overall, the latter study noted discordant viral populations between the blood and semen in 40% of individuals studied, regardless of whether or not they were co‐infected with another STI 34.

We have recently reexamined the relationship between HIV and STIs using single genome amplification followed by Sanger sequencing 32, as well as Primer ID 38, 39 and deep sequencing 40. Co‐infection with another STI did not appear to strongly influence the establishment of compartmentalized populations in this cohort, but individuals with urethritis tended to have more dynamic viral populations in the semen, than did men without urethritis 40.

Studies examining HIV‐1 diversity in the female genital tract during early infection have observed multiple variants not detected in the blood plasma 41. Multiple variants appear to be able to establish local foci of infection in the female genital tract, although perhaps only one or two are capable of initiating a disseminated infection. Subsequently, variable compartmentalization of HIV‐1 between the female genital tract and the blood has been observed. For example, Kemal *et al*. noted genotypically and phenotypically different HIV‐1 envelopes from viruses recovered from the female genital tract as compared with the blood 37. Phenotypic differences included the use of CXCR4 as a coreceptor and an increased number of N‐linked glycosylation sites. This observation, coupled with the fact that compartmentalized lineages were most often found in individuals with low CD4 counts, led to the hypothesis that local immune pressures in the female genital tract were driving viral evolution.

However, as PCR techniques and sequencing methods that limit recombination and resampling were developed, a different picture of compartmentalization in the female genital tract has emerged 35, 36. Although genetically distinct lineages are often found in the genital tract, they are most often monophyletic, indicative of short bursts of replication. Furthermore, when women were followed longitudinally for five years, no tissue‐specific phenotype persisted 36. While more work is needed, it appears that compartmentalization in the female genital tract may be a transient phenomenon. Longitudinal studies of compartmentalization in the male genital tract in the presence and absence of STIs are currently in progress, but a similar pattern of transient compartmentalization was observed in a small number of men who were followed for 180 days during acute and early infection 33.

### 2.4 STIs and susceptibility to HIV

Transmission of classical STIs is generally more efficient than HIV, and therefore may set the stage for increased risk of HIV acquisition 17. Inflammation and ulcers can be expected to lower the barrier(s) to infection 15, 42, 43. Recent studies have tried to more precisely define the conditions that lead to HIV acquisition in women, with a focus on unique cytokine profiles 15, 44 and disturbance of vaginal microbiome 45 with resultant “dysbiosis” (non‐optimal vaginal flora) 46. STIs can evoke an influx of receptive cells with expression of a greater number of CCR5 and CD4 receptors per cell 17. The risk of HIV acquisition for a woman with mucosal inflammation or a genital ulcer is greatly increased 17. Trichomonas infection in women, a common pathogen, also increases HIV acquisition 22. It should be noted that people with an STI appear to be susceptible to an HIV viral variant with reduced fitness 42.

The foreskin is a critical point of acquisition of HIV by men. It has been argued that low‐grade inflammation in this tissue, perhaps critical to decrease commensal bacterial colonization and to resist STIs, increases the risk of HIV acquisition 47, 48. Circumcision greatly decreases the risk of HIV infection 49. Circumcision also appears to reduce the risk of genital ulcer disease in men 47.

Rectal mucosa is a vulnerable tissue and unprotected anal intercourse has the greatest risk for HIV acquisition 3, 4, 5, 50. Rectal mucosa is thin and friable and heavily defended against infection, thereby enriched with cells receptive to HIV. Bernstein *et al*. reported that in men who have sex with men (MSM) with a history of syphilis and two rectal gonorrhoea or chlamydia infections in the past two years, there was an eightfold risk of HIV acquisition 51.

### 2.5 STIs and prevention trials

The role of STIs in the spread of HIV led to a series of randomized clinical trials designed to reduce the incidence of HIV infection in communities 52, 53, 54, 55, 56, 57, in individuals 58, 59 and in serodifferent couples 60, 61, 62. Of the nine clinical trials, successful prevention of HIV through treatment of STIs was only noted in Mwanza, Tanzania 52. The differing results of these trials have been extensively reviewed 16, 17, 61, 62. Failure to see population level prevention of HIV acquisition by more aggressive or mass treatment of STIs is best ascribed to the difficulty of providing effective drugs to the right people at the right time, and the difficulty of assuring that the trial participants are able to adhere to the antimicrobial regimens selected.

An alternative approach has been to focus on HSV‐2 treatment to prevent individual HIV acquisition 58, 59 or transmission 60. HSV‐2 was chosen as a key target because it is such a common infection and so strongly associated with HIV transmission 61, 63. Acyclovir was used to suppress HSV‐2 replication. No prevention benefit was observed whether the agent was used to treat HIV positive or negative people (the latter representing HSV‐2 PrEP). It seems likely that subclinical inflammation in spite of treatment reduced the anticipated benefit(s) of acyclovir 64. Mugwanya *et al*. 65 has reported that high‐dose valacyclovir (1.5 grams twice daily) might reduce HIV‐1 infectiousness more than acyclovir treatment used in earlier clinical trials.

### 2.6 STI biology in the era of ART

Several studies have shown that ART prevents secondary HIV transmission independent of STI coinfections 66, 67, 68, 69, 70, 71. In the HPTN 052 multinational randomized controlled trial, HIV transmission was virtually eliminated in HIV discordant heterosexual couples when viral replication was successfully suppressed 66, 69; STIs were commonly detected in study subjects over more than 10,000 person‐years of follow‐up. The latter results were confirmed by more recent observational cohort studies of both heterosexual and MSM couples 67, 68, 70, 71. The PARTNER study 67 followed HIV‐serodifferent couples reporting condomless sex and where the HIV‐infected partner was taking ART, during 1238 person‐years in 888 partnerships, no genetically linked HIV transmissions were detected when the HIV‐positive partner was virally suppressed, despite frequent incident STIs in the HIV‐positive partner (18% among MSM and 6% among heterosexual men and women) or negative partner (17% among MSM and 6% among heterosexual men and women). More recently, Rodger *et al*. reported that in a continuation of the Partner study, 779 MSM couples reported 76,088 episodes of condomless anal intercourse with no linked HIV transmission events 70, 71. In this study, 24% of HIV positive men and 27% of their HIV negative sexual partners acquired an STI. In the Opposites Attract study of serodifferent MSM couples 68), 1/3 of HIV‐positive participants and 1/4 of HIV‐negative participants acquired STIs during follow‐up, with an incidence rate of 22.8 STIs per 100 person‐years and 15.1 STIs per 100 person‐years respectively. However, no genetically linked HIV transmission events were documented during the 588.4 couple‐years of follow‐up 68.

### 2.7 Do STIs influence HIV‐1 shedding in spite of antiretroviral therapy?

However, while HIV treatment reliably prevents HIV transmission, it does not prevent shedding of the virus in the genital secretions of men 72 or women 73.

### 2.8 STIs and HIV in the female genital tract

There are a large series of reports of detection of HIV virus in the female genital tract with a wide variety of STIs 74, 75, 76. Graham and colleagues sought to understand how genital ulceration impacted cervical and vaginal shedding of HIV‐1 in women receiving ART in Kenya 77. Among 145 women who initiated ART, 36 developed a genital ulcer after at least two months of ART; ten women (28%) had detectable HIV‐1 RNA in their genital secretions. King and colleagues 78 followed 1114 women initiating ART to determine factors that influence viral shedding. During 5.8% of patient visits (among 76 women with 83 visits), HIV‐1 RNA was detected in genital secretions but not blood plasma. The median concentration of HIV‐1 RNA in genital secretions was between 1000 and 5000 copies/mL. As time on ART increased, the proportion of women with detectable genital HIV‐1 RNA decreased. Correlates of detectable HIV‐1 RNA in the genital tract in women with undetectable HIV in blood included more advanced WHO stage of disease, the presence of an ulcerative STI, cervical tenderness and the antiretroviral combination employed. The latter observation emphasizes differences in the pharmacology of ART in the male and female genital tract that can influence the suppression of replication of HIV 27, 79, 80, 81.

### 2.9 STIs and HIV in the male genital tract

Kalichman *et al*. studied the relationship between blood and seminal plasma, and shedding of HIV in semen in spite of ART 82. He reviewed studies demonstrating 100s and sometime 1,000s of copies of HIV‐1 RNA in semen when less than 50 copies of HIV were detected in blood. Anderson *et al*. reviewed the association between seminal cells and HIV transmission, and the possibility that ART may not eliminate cells that remain infectious 83. HIV virus can be detected in semen in 5‐30% samples obtained from men on ART 82, 84. It should be noted that different antiretroviral regimens may reduce HIV viral concentration in genital secretions with different speed and efficiency 80, 81; integrase inhibitors appear particularly effective in reducing HIV in semen 85.

Only a few studies of the effects of STIs on semen shedding in men receiving ART have been reported. Sadiq *et al*. studied the blood and seminal fluid of 24 men receiving ART who acquired urethritis 86. They reported two men (17%) with urethritis who had low blood viral loads at study screening with increased HIV viral loads in semen (5928 and 1512 copies HIV RNA/mL). The seminal viral loads reverted to <1000 copies HIV RNA/mL after STI treatment.

To further investigate the issue, we have enrolled HIV‐infected men with acute urethritis into an ongoing prospective observational cohort 87. Among 56 men enrolled in the study with at least 12 weeks of ART (<1000 copies/mL blood at baseline), nine subjects (16%) had HIV ≥1000 copies/mL detected in semen within the first two weeks of enrolment. HIV in semen was <1000 copies/mL within eight weeks of treatment for urethritis, consistent with an earlier study 18.

In men with acute urethritis who were not on ART at enrolment but initiated treatment within one week, HIV copy number in both blood and semen were comparable (baseline median viral loads of 4.7 and 4.1 log_10_ copies/mL respectively) 88. However, while both compartments showed decreasing viral loads after ART initiation, (week eight median viral loads of 2.0 and 0.0 log_10_ copies/mL in the blood and semen respectively); semen viral loads showed higher variability over time.

There is also little information to date about the effects of STIs on HIV viral shedding in the rectum. Kelley *et al*. examined the associations between rectal chlamydia and gonorrhoea, HSV‐2 seropositivity and HIV viral shedding, and found that STIs had little effect 89. Although these results were underpowered to stratify by ART use, 74% of the participants in the study were prescribed ART, and the results showed no effect of STI coinfection at low blood plasma viral loads of <1000 copies/mL. Davies *et al*. also assessed differences in rectal viral loads among MSM on ART with and without STIs 90. Among their 18 participants, they found no significant difference in rectal viral loads between those with and without STIs; all rectal viral loads from both STI groups were below the limit of detection 90.

The detection of HIV RNA and the DNA in the genital secretions evoked by an STI suggests escape of the virus (or some part of the virus) from the cell, or release of latent virus, or viral replication. However, failure of HIV‐positive people to infect their sexual partners 66, 67, 68, 69, 70 strongly suggests that viral copies detected are defective (and not replication competent) and/or that ART in the genital tract also contributes to HIV prevention. The majority of HIV viruses recovered from the latent pool in blood are defective and not replication competent 91, similar detailed studies have not yet been conducted with viral copies recovered from the genital tract.

### 2.10 STIs and blood HIV burden

A related question is the effects of STIs on blood viral burden**.** As noted above, genital ulcers significantly increase the amount of viral RNA shed in both the male 92 and female genital tracts 14. Buchaz *et al*. reported increased HIV in blood in people with primary and secondary syphilis 93. Dyer *et al*. 92 found an increase in blood viral burden in men with genital ulcers and urethritis. Celum *et al*. 60 found a modest reduction of HIV in blood from treatment of HSV‐2 with acyclovir. Lingappa *et al*. 94 reported that acyclovir could reduce progression of HIV disease in people dually infected with HIV and HSV‐2. These results suggest a systemic effect of HSV‐2 infection.

Antiretroviral therapy is highly effective at suppressing HIV‐1 replication in the blood, including in people with STIs. In a meta‐analysis of 14 studies looking at the effects of STI infection on HIV‐1 blood viral load, Champredon and colleagues concluded that co‐infection with an STI correlates with a 0.11 log_10_ increase in HIV‐1 viral load suggesting that when an individual is suppressed on ART, STIs have little effect on blood viral load 95.

### 2.11 STIs and pre‐exposure prophylaxis in MSM

A series of clinical trials demonstrated that TDF/FTC can prevent HIV acquisition in MSM 96, 97, 98 and women [reviewed in 99].

TDF/FTC prophylaxis was approved by the US CDC in 2012 and guidelines are available 100. However, one major concern has been the effects of pre‐exposure prophylaxis (PrEP) on sexual behaviours that might lead to an STI. In a systematic review of 17 open label PrEP studies with meta‐analysis of eight studies that included measurement of STIs, Traeger *et al*. noted a modest increase (odds ratio 1.24, 95% CI: 0.99‐1.54) in STI risk associated with TDF/FTC PrEP, especially in more recent studies 101. However, another meta‐analysis estimated that among MSM taking TDF/FTC PrEP, the incidence rates for gonorrhoea, chlamydia and syphilis were 25.3, 11.2 and 44.6 times the incidence rates among MSM not taking PrEP 102. Although both results suggest increased risk of STIs among men taking TDF/FTC PrEP, the relative strengths of the associations reported were quite dissimilar. As noted in the respective studies and further commentary 103, selection of high‐risk participants into PrEP studies and decreased STI detection among non‐PrEP users may have biased some results upwards.

Most recently, Traeger *et al*. 104 prospectively evaluated incidence of chlamydia, gonorrhoea and syphilis in 2891 MSM and bisexual men enrolled in a PrEP trial in Victoria, Australia. The authors noted significant increases in STIs over 1.1 years of follow‐up. However, 76% of STIs were noted in only 736 of the study participants. In addition, increases in STIs were not associated with decreased condom usage, although condom usage was not always consistent, and condoms were probably not used during oral‐penile sex when some pathogens could be transmitted 105. The investigators suggested that changes in sexual networks or sexual behaviours in some PrEP users might lead to increases in STIs. They found risk factors predicting an incident STI in men receiving TDF/FTC PrEP to include younger age, greater partner number and group sex. The results support the frequent measurement and treatment of STIs in PrEP users 100.

A second critical question is the efficacy of TDF/FTC PrEP when an STI is acquired. This question was addressed in an observational report from Kaiser Permanente California 106. Among 687 men who initiated TDF/FTC PrEP, 187 acquired STIs; however, no incident cases of HIV acquisition were noted. In recent prospective clinical trials in MSM—IPERGAY, and Proud—TDF/FTC PrEP prevented 86% and 96% respectively, of HIV infections regardless of high incidence of STI infections during the trials 97, 98. In the IPERGAY trial 98, 43% of MSM randomized to the PrEP arm acquired one or more STIs. In the Proud study 97, 57% of study subjects receiving PrEP had an STI and 36% had rectal gonorrhoea or chlamydia. These results convincingly demonstrate that incident STIs do not compromise the prevention benefit of TDF/FTC PrEP in MSM. However, as new PrEP drugs are developed (see below) each agent must independently demonstrate the ability to withstand the inflammatory changes evoked by an STI.

### 2.12 STIs and PrEP in women

PrEP effectiveness in either partner in serodifferent heterosexual couples 107, and in HIV‐negative women 107, 108, 109, 110, 111, 112, 113, has also been examined but with mixed results. The Partners PrEP and TDF2 studies both found significant reductions in HIV acquisition among men and women using oral TDF 107 or TDF/FTC 107, 111 in Sub‐Saharan Africa. The CAPRISA study found modest reduction in HIV acquisition among women in Sub‐Saharan Africa with 1% vaginal gel formulation of a tenofovir topical microbicide 108, as did trials using a dapivirine vaginal ring microbicide 109, 114.

The VOICE trial 110, which evaluated oral TDF, oral TDF/FTC and 1% tenofovir vaginal gel, and the FEM‐PrEP trial 112, which evaluated oral TDF/FTC failed to find significant reductions in HIV‐acquisition among women in Sub‐Saharan Africa. For the most part, these differences have been ascribed to limited adherence to PrEP products, including topical microbicides. However, it is possible that one or more concomitant STIs compromise the efficacy of oral or topical PrEP in women 113. Indeed, McKinnon *et al*. 48 reported that genital inflammation reduced the efficacy of tenofovir gel.

### 2.13 New PrEP drugs and STIs

Most recently the results of a clinical trial that directly compared TDF/FTC with tenofovir alfenamide TAF and FTC demonstrated the equivalency of the latter combination, although very few incident infections were detected 115. As an alternative to oral PrEP the integrase strand inhibitor cabotegravir has potential for long acting PrEP 116. Landovitz *et al*. identified a dose and dosage schedule for cabotegravir as PrEP 116. This agent is now being compared directly to TDF/FTC daily and TDF/FTC every eight weeks injection in more than 5000 high risk men and women (NCT02720094; NCT03164564). An important consideration is the HIV prevention efficacy of cabotegravir in the presence of an STI, and this is being explored. There is considerable interest in other means of delivering long acting HIV prevention in vaginal rings 109, 114, or implants 117 or microneedle patches 118. These devices could potentially combine HIV and STI prevention, and contraception into a “multipurpose intervention.”

### 2.14 Mathematical modelling

Mathematical modelling has been used to understand the spread of HIV and compare prevention strategies 119, 120, 121. Such combination interventions generally include voluntary male circumcision, behaviour change (which generally includes emphasis on detection and treatment of STIs), and ART used as “treatment as prevention” (TaSP) or PrEP. Chesson and coworkers have argued that gonorrhoea, chlamydia and syphilis contribute to the HIV epidemic, and that their treatment may be a cost effective way to reduce the spread of HIV 122, 123. However, as indicated above, mass treatment of STIs did not have the benefits anticipated in these models. These results demonstrate the difficulty of treating bacterial and viral STIs, and the concern that STIs may reflect risk behaviours and exposure to HIV rather than (or at least as much as) serving to amplify HIV transmission.

Jenness *et al*. 123 have suggested a unique benefit of PrEP for MSM in the United States and perhaps other high‐income settings. In their model they propose that adherence to CDC PrEP guidelines 100 would increase STI screening so much that 42% of gonorrhoeal infections, and 40% of chlamydial infections could be prevented over the next decade 123.

### 2.15 PrEP for STIs

As already noted, HIV PrEP trials have found high incidence of classical STIs 96, 97, 98, 104. Bolan *et al*. 124 and Molina *et al*. 125 have reported the successful use of doxycycline prophylaxis to reduce the incidence of syphilis and chlamydia in high risk MSM. Doxycycline was not effective for prevention of gonorrhoea. These results further emphasize the importance of consideration of STIs in the treatment and prevention of HIV infection.

## 3 Conclusions

The early history of the HIV pandemic was marked by realization that HIV infection led to a new, fatal sexually transmitted disease with risk to both sexually active men and women, and that several classical STIs amplified both infectiousness and acquisition of HIV 10, 13, 17. While all STIs can and do occur concomitantly, the influence of classical STIs on HIV transmission is unique. Emphasis on this relationship led to attempts to reduce HIV incidence through more STI testing and treatment. But failure of mass treatment to reduce HIV infection in most clinical trials 61 reduced the interest of the HIV research community in STIs, and perhaps reduced funding for detection and treatment of STIs. Sadly, a wide variety of factors have accelerated spread of STIs, especially among MSM at high risk for HIV acquisition 11. Particularly severe problems with syphilis infections and increasing resistance of gonorrhoea to antibiotics have been emphasized 11.

Where do we go from here? We have no choice but to rethink STI research goals and intervention funding, and the relationship between STIs and HIV; and new questions have arisen. We do not understand the biology of shedding of HIV in the genital tract that persists despite clearance in the blood with ART. This problem is highly relevant to thinking about the cure of HIV. Several strategies are now being pursued to permit remission (no drugs required) or sterilising cure of HIV. Among the most popular is the “kick and kill” strategy with reactivation of latent HIV virus and concomitant elimination of HIV infected cells 126, 127. The increased shedding of HIV in the genital tract evoked by some STI infections demonstrates the well documented compartmentalization of HIV. In this case, STIs are acting as a “kick.” So lessons learned about the effects of shedding of HIV in the genital tract are highly relevant and perhaps critical to the ultimate cure of the infection. This situation also draws attention to the need for better understanding of the pharmacology of antiretroviral agents in the genital tract 80, 81. However, viral copies detected in the genital tract under these conditions do not lead to HIV transmission.

Finally, there is the complex and evolving relationship between PrEP, STIs and HIV acquisition. Currently, the only agent approved in the US as pre‐exposure prophylaxis (PrEP) is the fixed dose combination of tenofovir disoproxil fumarate and emtricitabine (TDF/FTC). The use of TDF/FTC has been accompanied by recognition of high incidence of STIs in PrEP users 96, 97, 98, 104, 106. Sexual risk behaviours that preceded availability of PrEP and increased post PrEP risk behaviours (from reduced fear of HIV because of excellent treatment of HIV and PrEP or other social forces) have been convincingly demonstrated 11. But fortunately, STIs do not increase HIV acquisition in people using TDF/FTC PrEP; importantly, the prevention benefit of TDF/FTC is not overwhelmed by ulcers or inflammation. However, for each new PrEP agent, such as with tenofovir alafenamide (TAF) (Discover, NCT02842086) 115, or cabotegravir LA (an injectable long acting integrase inhibitor, HPTN 083, NCT02720094, HPTN 084, NCT03164564) or one or more broad neutralizing antibodies 128, we must prove that the prevention benefit persists in the presence of one or more STIs.

STIs are a harbinger of HIV acquisition, depending on the prevalence of HIV in the community, the number of people on treatment, and the degree of difficulty in detection and treatment of STIs and HIV. STIs serve as a critical surrogate for the need for PrEP 100, 129, and they represent a critical problem by themselves, a fact that is sometimes overlooked in public health funding decisions. STIs have critical consequences for sexual and reproductive health of men and women 11. The important and rapidly evolving STI pandemic will affect the spread and control of HIV. The relationship between STIs and HIV has been demonstrated over and over and over again during the past 30 years and this “synergy” 13 will not just go away; STIs must be urgently addressed with new ideas and increase in resources.

## Competing interests

MSC is on the Advisory Board for Merck and Gilead. ODC and JSC have no potential conflicts.

## Authors’ contributions

MSC provided conception and design, as well as analysis and interpretation of data; drafted manuscript, provided critical revisions and gave final approval of submission. ODC participated in drafting the article and critically revising for intellectual content. JSC participated in drafting the article and critically revising for intellectual content.

## References

[1] Royce RA , Sena A , Cates W Jr , Cohen MS . Sexual transmission of HIV. N Engl J Med. 1997 Apr;336(15):1072–8. 9091805 10.1056/NEJM199704103361507

[2] Boily MC , Baggaley RF , Wang L , Masse B , White RG , Hayes RJ , et al. Heterosexual risk of HIV‐1 infection per sexual act: systematic review and meta‐analysis of observational studies. Lancet Infect Dis. 2009;9(2):118–29. 19179227 10.1016/S1473-3099(09)70021-0 PMC4467783

[3] Powers KA , Poole C , Pettifor AE , Cohen MS . Rethinking the heterosexual infectivity of HIV‐1: a systematic review and meta‐analysis. Lancet Infect Dis. 2008;8(9):553–63. 18684670 10.1016/S1473-3099(08)70156-7 PMC2744983

[4] Baggaley RF , Owen BN , Silhol R , Elmes J , Anton P , McGowan I , et al. Does per‐act HIV‐1 transmission risk through anal sex vary by gender? An updated systematic review and meta‐analysis. Am J Reprod Immunol. 2018;80(5):e13039. 30175479 10.1111/aji.13039 PMC6202169

[5] Baggaley RF , White RG , Boily MC . HIV transmission risk through anal intercourse: systematic review, meta‐analysis and implications for HIV prevention. Int J Epidemiol. 2010;39(4):1048–63. 20406794 10.1093/ije/dyq057 PMC2929353

[6] Keele BF , Giorgi EE , Salazar‐Gonzalez JF , Decker JM , Pham KT , Salazar MG , et al. Identification and characterization of transmitted and early founder virus envelopes in primary HIV‐1 infection. Proc Natl Acad Sci USA. 2008;105(21):7552–7. 18490657 10.1073/pnas.0802203105 PMC2387184

[7] Tully DC , Ogilvie CB , Batorsky RE , Bean DJ , Power KA , Ghebremichael M , et al. Differences in the Selection Bottleneck between Modes of Sexual Transmission Influence the Genetic Composition of the HIV‐1 Founder Virus. PLoS Pathog. 2016;12(5):e1005619. 27163788 10.1371/journal.ppat.1005619 PMC4862634

[8] Fideli US , Allen SA , Musonda R , Trask S , Hahn BH , Weiss H , et al. Virologic and immunologic determinants of heterosexual transmission of human immunodeficiency virus type 1 in Africa. AIDS Res Hum Retroviruses. 2001;17(10):901–10. 11461676 10.1089/088922201750290023 PMC2748905

[9] Quinn TC , Wawer MJ , Sewankambo N , Serwadda D , Li C , Wabwire‐Mangen F , et al. Viral load and heterosexual transmission of human immunodeficiency virus type 1. Rakai Project Study Group. N Engl J Med. 2000;342(13):921–9. 10738050 10.1056/NEJM200003303421303

[10] Cohen M . Amplified transmission of HIV‐1: New clues to the AIDS pandemic. Tran Am Clin Climatol Assoc. 2006;117:213–25. PMC1500941 18528475

[11] Unemo M , Bradshaw CS , Hocking JS , de Vries HJC , Francis SC , Mabey D , et al. Sexually transmitted infections: challenges ahead. Lancet Infect Dis. 2017;17(8):e235–79. 28701272 10.1016/S1473-3099(17)30310-9

[12] Piot P , Laga M . Genital ulcers, other sexually transmitted diseases, and the sexual transmission of HIV. BMJ. 1989;298(6674):623–4. 2496785 10.1136/bmj.298.6674.623 PMC1835849

[13] Wasserheit JN . Epidemiological synergy. Interrelationships between human immunodeficiency virus infection and other sexually transmitted diseases. Sex Transm Dis. 1992;19(2):61–77. 1595015

[14] Ghys PD , Fransen K , Diallo MO , Ettiegne‐Traore V , Coulibaly IM , Yeboue KM , et al. The associations between cervicovaginal HIV shedding, sexually transmitted diseases and immunosuppression in female sex workers in Abidjan, Cote d'Ivoire. AIDS. 1997;11(12):F85–93. 9342059 10.1097/00002030-199712000-00001

[15] Passmore JA , Jaspan HB , Masson L . Genital inflammation, immune activation and risk of sexual HIV acquisition. Curr Opin HIV AIDS. 2016;11(2):156–62. 26628324 10.1097/COH.0000000000000232 PMC6194860

[16] Mayer KH , Venkatesh KK . Interactions of HIV, other sexually transmitted diseases, and genital tract inflammation facilitating local pathogen transmission and acquisition. Am J Reprod Immunol. 2011;65(3):308–16. 21214660 10.1111/j.1600-0897.2010.00942.x PMC3077541

[17] Galvin SR , Cohen MS . The role of sexually transmitted diseases in HIV transmission. Nat Rev Microbiol. 2004;2(1):33–42. 15035007 10.1038/nrmicro794

[18] Cohen MS , Hoffman IF , Royce RA , Kazembe P , Dyer JR , Daly CC , et al. Reduction of concentration of HIV‐1 in semen after treatment of urethritis: implications for prevention of sexual transmission of HIV‐1. AIDSCAP Malawi Research Group. Lancet. 1997;349(9069):1868–73. 9217758 10.1016/s0140-6736(97)02190-9

[19] Price MA , Zimba D , Hoffman IF , Kaydos‐Daniels SC , Miller WC , Martinson F , et al. Addition of treatment for trichomoniasis to syndromic management of urethritis in Malawi: a randomized clinical trial. Sex Transm Dis. 2003;30(6):516–22. 12782954 10.1097/00007435-200306000-00009

[20] Gianella S , Smith DM , Vargas MV , Little SJ , Richman DD , Daar ES , et al. Shedding of HIV and human herpesviruses in the semen of effectively treated HIV‐1‐infected men who have sex with men. Clin Infect Dis. 2013;57(3):441–7. 23595831 10.1093/cid/cit252 PMC3703105

[21] Wang CC , McClelland RS , Reilly M , Overbaugh J , Emery SR , Mandaliya K , et al. The effect of treatment of vaginal infections on shedding of human immunodeficiency virus type 1. J Infect Dis. 2001;183(7):1017–22. 11237825 10.1086/319287

[22] Kissinger P , Adamski A . Trichomoniasis and HIV interactions: a review. Sex Transm Infect. 2013;89(6):426–33. 23605851 10.1136/sextrans-2012-051005 PMC3748151

[23] Johnson LF , Lewis DA . The effect of genital tract infections on HIV‐1 shedding in the genital tract: a systematic review and meta‐analysis. Sex Transm Dis. 2008;35(11):946–59. 18685546 10.1097/OLQ.0b013e3181812d15

[24] Cohen CR , Lingappa JR , Baeten JM , Ngayo MO , Spiegel CA , Hong T , et al. Bacterial vaginosis associated with increased risk of female‐to‐male HIV‐1 transmission: a prospective cohort analysis among African couples. PLoS Med. 2012;9(6):e1001251. 22745608 10.1371/journal.pmed.1001251 PMC3383741

[25] Baeten JM , Kahle E , Lingappa JR , Coombs RW , Delany‐Moretlwe S , Nakku‐Joloba E , et al. Genital HIV‐1 RNA predicts risk of heterosexual HIV‐1 transmission. Sci Transl Med. 2011;3(77):77ra29. 10.1126/scitranslmed.3001888 PMC3087186 21471433

[26] Maldarelli F , Kearney M , Palmer S , Stephens R , Mican J , Polis MA , et al. HIV populations are large and accumulate high genetic diversity in a nonlinear fashion. J Virol. 2013;87(18):10313–23. 23678164 10.1128/JVI.01225-12 PMC3754011

[27] Joseph SB , Swanstrom R , Kashuba AD , Cohen MS . Bottlenecks in HIV‐1 transmission: insights from the study of founder viruses. Nat Rev Microbiol. 2015;13(7):414–25. 26052661 10.1038/nrmicro3471 PMC4793885

[28] Goonetilleke N , Liu MK , Salazar‐Gonzalez JF , Ferrari G , Giorgi E , Ganusov VV , et al. The first T cell response to transmitted/founder virus contributes to the control of acute viremia in HIV‐1 infection. J Exp Med. 2009;206(6):1253–72. 19487423 10.1084/jem.20090365 PMC2715063

[29] Barton JP , Goonetilleke N , Butler TC , Walker BD , McMichael AJ , Chakraborty AK . Relative rate and location of intra‐host HIV evolution to evade cellular immunity are predictable. Nat Commun. 2016;7:11660. 27212475 10.1038/ncomms11660 PMC4879252

[30] Chaillon A , Gianella S , Wertheim JO , Richman DD , Mehta SR , Smith DM . HIV migration between blood and cerebrospinal fluid or semen over time. J Infect Dis. 2014;209(10):1642–52. 24302756 10.1093/infdis/jit678 PMC3997580

[31] Sturdevant CB , Joseph SB , Schnell G , Price RW , Swanstrom R , Spudich S . Compartmentalized replication of R5 T cell‐tropic HIV‐1 in the central nervous system early in the course of infection. PLoS Pathog. 2015;11(3):e1004720. 25811757 10.1371/journal.ppat.1004720 PMC4374811

[32] Anderson JA , Ping LH , Dibben O , Jabara CB , Arney L , Kincer L , et al. HIV‐1 Populations in Semen Arise through Multiple Mechanisms. PLoS Pathog. 2010;6(8):e1001053. 20808902 10.1371/journal.ppat.1001053 PMC2924360

[33] Chaillon A , Smith DM , Vanpouille C , Lisco A , Jordan P , Caballero G , et al. HIV Trafficking Between Blood and Semen During Early Untreated HIV Infection. J Acquir Immune Defic Syndr. 2017;74(1):95–102. 27548440 10.1097/QAI.0000000000001156 PMC5140710

[34] Ping LH , Cohen MS , Hoffman I , Vernazza P , Seillier‐Moiseiwitsch F , Chakraborty H , et al. Effects of genital tract inflammation on human immunodeficiency virus type 1 V3 populations in blood and semen. J Virol. 2000;74(19):8946–52. 10982338 10.1128/jvi.74.19.8946-8952.2000 PMC102090

[35] Bull M , Learn G , Genowati I , McKernan J , Hitti J , Lockhart D , et al. Compartmentalization of HIV‐1 within the female genital tract is due to monotypic and low‐diversity variants not distinct viral populations. PLoS ONE. 2009;4(9):e7122. 19771165 10.1371/journal.pone.0007122 PMC2741601

[36] Bull ME , Heath LM , McKernan‐Mullin JL , Kraft KM , Acevedo L , Hitti JE , et al. Human immunodeficiency viruses appear compartmentalized to the female genital tract in cross‐sectional analyses but genital lineages do not persist over time. J Infect Dis. 2013;207(8):1206–15. 23315326 10.1093/infdis/jit016 PMC3603533

[37] Kemal KS , Foley B , Burger H , Anastos K , Minkoff H , Kitchen C , et al. HIV‐1 in genital tract and plasma of women: compartmentalization of viral sequences, coreceptor usage, and glycosylation. Proc Natl Acad Sci USA. 2003;100(22):12972–7. 14557540 10.1073/pnas.2134064100 PMC240729

[38] Jabara CB , Jones CD , Roach J , Anderson JA , Swanstrom R . Accurate sampling and deep sequencing of the HIV‐1 protease gene using a Primer ID. Proc Natl Acad Sci USA. 2011;108(50):20166–71. 22135472 10.1073/pnas.1110064108 PMC3250168

[39] Keys JR , Zhou S , Anderson JA , Eron JJ Jr , Rackoff LA , Jabara C , et al. Primer ID Informs Next‐Generation Sequencing Platforms and Reveals Preexisting Drug Resistance Mutations in the HIV‐1 Reverse Transcriptase Coding Domain. AIDS Res Hum Retroviruses. 2015;31(6):658–68. 25748056 10.1089/aid.2014.0031 PMC4458739

[40] Council O , Ping L , McCann C , Hoffman I , Tegha G , Kamwendo D , et al. Compartmentalized HIV‐1 is found in the semen of men with and without urethritis. Conference on Retroviruses and Opportunistic Infections (CROI), Boston, MA; 2018.

[41] Klein K , Nickel G , Nankya I , Kyeyune F , Demers K , Ndashimye E , et al. Higher sequence diversity in the vaginal tract than in blood at early HIV‐1 infection. PLoS Pathog. 2018;14(1):e1006754. 29346424 10.1371/journal.ppat.1006754 PMC5773221

[42] Carlson JM , Schaefer M , Monaco DC , Batorsky R , Claiborne DT , Prince J , et al. HIV transmission. Selection bias at the heterosexual HIV‐1 transmission bottleneck. Science. 2014;345(6193):1254031. 25013080 10.1126/science.1254031 PMC4289910

[43] Masson L , Passmore JA , Liebenberg LJ , Werner L , Baxter C , Arnold KB , et al. Genital inflammation and the risk of HIV acquisition in women. Clin Infect Dis. 2015;61(2):260–9. 25900168 10.1093/cid/civ298 PMC4565995

[44] Liebenberg LJ , Masson L , Arnold KB , McKinnon LR , Werner L , Proctor E , et al. Genital‐systemic chemokine gradients and the risk of HIV acquisition in women. J Acquir Immune Defic Syndr. 2017;74(3):318–25. 28187085 10.1097/QAI.0000000000001218 PMC5305292

[45] McClelland RS , Lingappa JR , Srinivasan S , Kinuthia J , John‐Stewart GC , Jaoko W , et al. Evaluation of the association between the concentrations of key vaginal bacteria and the increased risk of HIV acquisition in African women from five cohorts: a nested case‐control study. Lancet Infect Dis. 2018;18(5):554–64. 29396006 10.1016/S1473-3099(18)30058-6 PMC6445552

[46] McKinnon LR , Achilles SL , Bradshaw CS , Burgener A , Crucitti T , Fredricks DN , et al. The evolving facets of bacterial vaginosis: implications for HIV transmission. AIDS Res Hum Retroviruses. 2019;35(3):219–28. 30638028 10.1089/aid.2018.0304 PMC6434601

[47] Esra RT , Olivier AJ , Passmore JA , Jaspan HB , Harryparsad R , Gray CM . Does HIV exploit the inflammatory milieu of the male genital tract for successful infection? Front Immunol. 2016;7:245. 27446076 10.3389/fimmu.2016.00245 PMC4919362

[48] McKinnon LR , Liebenberg LJ , Yende‐Zuma N , Archary D , Ngcapu S , Sivro A , et al. Genital inflammation undermines the effectiveness of tenofovir gel in preventing HIV acquisition in women. Nat Med. 2018;24(4):491–6. 29480895 10.1038/nm.4506 PMC5893390

[49] World Health Organization, Joint United Nations Programme on HIV and AIDS . WHO and UNAIDS Announce Recommendations from Expert Consultation on Male Circumcision for HIV Prevention. Montreux, Switzerland: World Health Organization Vol. 1. 2007.

[50] Patel P , Borkowf CB , Brooks JT , Lasry A , Lansky A , Mermin J . Estimating per‐act HIV transmission risk: a systematic review. AIDS. 2014;28(10):1509–19. 24809629 10.1097/QAD.0000000000000298 PMC6195215

[51] Bernstein KT , Marcus JL , Nieri G , Philip SS , Klausner JD . Rectal gonorrhea and chlamydia reinfection is associated with increased risk of HIV seroconversion. J Acquir Immune Defic Syndr. 2010;53(4):537–43. 19935075 10.1097/QAI.0b013e3181c3ef29

[52] Grosskurth H , Mosha F , Todd J , Mwijarubi E , Klokke A , Senkoro K , et al. Impact of improved treatment of sexually transmitted diseases on HIV infection in rural Tanzania: randomised controlled trial. Lancet. 1995;346(8974):530–6. 7658778 10.1016/s0140-6736(95)91380-7

[53] Wawer MJ , Sewankambo NK , Serwadda D , Quinn TC , Paxton LA , Kiwanuka N , et al. Control of sexually transmitted diseases for AIDS prevention in Uganda: a randomised community trial. Rakai Project Study Group. Lancet. 1999;353(9152):525–35. 10028980 10.1016/s0140-6736(98)06439-3

[54] Ghys PD , Diallo MO , Ettiegne‐Traore V , Satten GA , Anoma CK , Maurice C , et al. Effect of interventions to control sexually transmitted disease on the incidence of HIV infection in female sex workers. AIDS. 2001;15(11):1421–31. 11504964 10.1097/00002030-200107270-00012

[55] Gregson S , Adamson S , Papaya S , Mundondo J , Nyamukapa CA , Mason PR , et al. Impact and process evaluation of integrated community and clinic‐based HIV‐1 control: a cluster‐randomised trial in eastern Zimbabwe. PLoS Med. 2007;4(3):e102. 17388666 10.1371/journal.pmed.0040102 PMC1831737

[56] Kamali A , Quigley M , Nakiyingi J , Kinsman J , Kengeya‐Kayondo J , Gopal R , et al. Syndromic management of sexually‐transmitted infections and behaviour change interventions on transmission of HIV‐1 in rural Uganda: a community randomised trial. Lancet. 2003;361(9358):645–52. 12606175 10.1016/s0140-6736(03)12598-6

[57] Kaul R , Kimani J , Nagelkerke NJ , Fonck K , Ngugi EN , Keli F , et al. Monthly antibiotic chemoprophylaxis and incidence of sexually transmitted infections and HIV‐1 infection in Kenyan sex workers: a randomized controlled trial. JAMA. 2004;291(21):2555–62. 15173146 10.1001/jama.291.21.2555

[58] Watson‐Jones D , Weiss HA , Rusizoka M , Changalucha J , Baisley K , Mugeye K , et al. Effect of herpes simplex suppression on incidence of HIV among women in Tanzania. N Engl J Med. 2008;358(15):1560–71. 18337596 10.1056/NEJMoa0800260 PMC2643126

[59] Celum C , Wald A , Hughes J , Sanchez J , Reid S , Delany‐Moretlwe S , et al. Effect of aciclovir on HIV‐1 acquisition in herpes simplex virus 2 seropositive women and men who have sex with men: a randomised, double‐blind, placebo‐controlled trial. Lancet. 2008;371(9630):2109–19. 18572080 10.1016/S0140-6736(08)60920-4 PMC2650104

[60] Celum C , Wald A , Lingappa JR , Magaret AS , Wang RS , Mugo N , et al. Acyclovir and transmission of HIV‐1 from persons infected with HIV‐1 and HSV‐2. N Engl J Med. 2010;362(5):427–39. 20089951 10.1056/NEJMoa0904849 PMC2838503

[61] Hayes R , Watson‐Jones D , Celum C , van de Wijgert J , Wasserheit J . Treatment of sexually transmitted infections for HIV prevention: end of the road or new beginning? AIDS. 2010;24 Suppl 4:S15–26. 10.1097/01.aids.0000390704.35642.47 PMC3827743 21042049

[62] Wetmore CM , Manhart LE , Wasserheit JN . Randomized controlled trials of interventions to prevent sexually transmitted infections: learning from the past to plan for the future. Epidemiol Rev. 2010;32:121–36. 20519264 10.1093/epirev/mxq010 PMC2912604

[63] Wald A , Link K . Risk of human immunodeficiency virus infection in herpes simplex virus type 2‐seropositive persons: a meta‐analysis. J Infect Dis. 2002;185(1):45–52. 11756980 10.1086/338231

[64] Zhu J , Hladik F , Woodward A , Klock A , Peng T , Johnston C , et al. Persistence of HIV‐1 receptor‐positive cells after HSV‐2 reactivation is a potential mechanism for increased HIV‐1 acquisition. Nat Med. 2009;15(8):886–92. 19648930 10.1038/nm.2006 PMC2723183

[65] Mugwanya K , Baeten JM , Mugo NR , Irungu E , Ngure K , Celum C . High‐dose valacyclovir HSV‐2 suppression results in greater reduction in plasma HIV‐1 levels compared with standard dose acyclovir among HIV‐1/HSV‐2 coinfected persons: a randomized, crossover trial. J Infect Dis. 2011;204(12):1912–7. 21998479 10.1093/infdis/jir649 PMC3247811

[66] Cohen MS , Chen YQ , McCauley M , Gamble T , Hosseinipour MC , Kumarasamy N , et al. Prevention of HIV‐1 infection with early antiretroviral therapy. N Engl J Med. 2011;365(6):493–505. 21767103 10.1056/NEJMoa1105243 PMC3200068

[67] Rodger AJ , Cambiano V , Bruun T , Vernazza P , Collins S , van Lunzen J , et al. Sexual activity without condoms and risk of HIV transmission in serodifferent couples when the hiv‐positive partner is using suppressive antiretroviral therapy. JAMA. 2016;316(2):171–81. 27404185 10.1001/jama.2016.5148

[68] Bavinton BR , Pinto AN , Phanuphak N , Grinsztejn B , Prestage GP , Zablotska‐Manos IB , et al. Viral suppression and HIV transmission in serodiscordant male couples: an international, prospective, observational, cohort study. Lancet HIV. 2018;5(8):e438–47. 30025681 10.1016/S2352-3018(18)30132-2

[69] Cohen MS , Chen YQ , McCauley M , Gamble T , Hosseinipour MC , Kumarasamy N , et al. Antiretroviral therapy for the prevention of HIV‐1 transmission. N Engl J Med. 2016;375(9):830–9. 27424812 10.1056/NEJMoa1600693 PMC5049503

[70] Rodger A , Cambiano V , Bruun T , Vernazza P , Collins S , Corbelli GM , et al. Risk of HIV transmission through condomless sex in MSM couples with suppressive ART: The PARTNER2 Study extended results in gay men. 22nd International AIDS Conference; July 23‐27, 2018; Amsterdam, the Netherlands.

[71] Rodger A , Cambiano V , Bruun T , Vernazza P , Collins S , Degen O , et al. HIV transmission risk through condomless sex in gay couples with virally suppressive art: final results of the partner study. Lancet. 2019;393(10189):2428–38. 31056293 10.1016/S0140-6736(19)30418-0 PMC6584382

[72] Pasquier C , Walschaerts M , Raymond S , Moinard N , Saune K , Daudin M , et al. Patterns of residual HIV‐1 RNA shedding in the seminal plasma of patients on effective antiretroviral therapy. Basic Clin Androl. 2017;27:17. 28904798 10.1186/s12610-017-0063-x PMC5590187

[73] Cu‐Uvin S , DeLong AK , Venkatesh KK , Hogan JW , Ingersoll J , Kurpewski J , et al. Genital tract HIV‐1 RNA shedding among women with below detectable plasma viral load. AIDS. 2010;24(16):2489–97. 20736815 10.1097/QAD.0b013e32833e5043

[74] Fastring DR , Amedee A , Gatski M , Clark RA , Mena LA , Levison J , et al. Co‐occurrence of Trichomonas vaginalis and bacterial vaginosis and vaginal shedding of HIV‐1 RNA. Sex Transm Dis. 2014;41(3):173–9. 24521723 10.1097/OLQ.0000000000000089

[75] Sha BE , Zariffard MR , Wang QJ , Chen HY , Bremer J , Cohen MH , et al. Female genital‐tract HIV load correlates inversely with Lactobacillus species but positively with bacterial vaginosis and Mycoplasma hominis. J Infect Dis. 2005;191(1):25–32. 15592999 10.1086/426394

[76] Wessman M , Thorsteinsson K , Jensen JS , Storgaard M , Ronsholt FF , Johansen IS , et al. Bacterial vaginosis, human papilloma virus and herpes viridae do not predict vaginal HIV RNA shedding in women living with HIV in Denmark. BMC Infect Dis. 2017;17(1):376. 28569142 10.1186/s12879-017-2477-7 PMC5452403

[77] Graham SM , Masese L , Gitau R , Richardson BA , Mandaliya K , Peshu N , et al. Genital ulceration does not increase HIV‐1 shedding in cervical or vaginal secretions of women taking antiretroviral therapy. Sex Transm Infect. 2011;87(2):114–7. 20980464 10.1136/sti.2010.045369 PMC3081651

[78] King CC , Ellington SR , Davis NL , Coombs RW , Pyra M , Hong T , et al. Prevalence, magnitude, and correlates of HIV‐1 genital shedding in women on antiretroviral therapy. J Infect Dis. 2017;216(12):1534–40. 29240922 10.1093/infdis/jix550 PMC5853287

[79] Patterson KB , Prince HA , Kraft E , Jenkins AJ , Shaheen NJ , Rooney JF , et al. Penetration of tenofovir and emtricitabine in mucosal tissues: implications for prevention of HIV‐1 transmission. Sci Transl Med. 2011;3(112):112re4. 10.1126/scitranslmed.3003174 PMC3483088 22158861

[80] Thompson CG , Cohen MS , Kashuba AD . Antiretroviral pharmacology in mucosal tissues. J Acquir Immune Defic Syndr. 2013;63 Suppl 2:S240–7. 23764642 10.1097/QAI.0b013e3182986ff8 PMC3951793

[81] Trezza CR , Kashuba AD . Pharmacokinetics of antiretrovirals in genital secretions and anatomic sites of HIV transmission: implications for HIV prevention. Clin Pharmacokinet. 2014;53(7):611–24. 24859035 10.1007/s40262-014-0148-z PMC4094112

[82] Kalichman SC , Di Berto G , Eaton L . Human immunodeficiency virus viral load in blood plasma and semen: review and implications of empirical findings. Sex Transm Dis. 2008;35(1):55–60. 18217225 10.1097/olq.0b013e318141fe9b

[83] Anderson DJ , Politch JA , Nadolski AM , Blaskewicz CD , Pudney J , Mayer KH . Targeting Trojan horse leukocytes for HIV prevention. AIDS. 2010;24(2):163–87. 20010071 10.1097/QAD.0b013e32833424c8 PMC4097178

[84] Pomerantz RJ . Residual HIV‐1 infection during antiretroviral therapy: the challenge of viral persistence. AIDS. 2001;15(10):1201–11. 11426065 10.1097/00002030-200107060-00002

[85] Imaz A , Martinez‐Picado J , Niubo J , Kashuba AD , Ferrer E , Ouchi D , et al. HIV‐1‐RNA decay and dolutegravir concentrations in semen of patients starting a first antiretroviral regimen. J Infect Dis. 2016;214(10):1512–9. 27578849 10.1093/infdis/jiw406 PMC5091371

[86] Sadiq ST , Taylor S , Kaye S , Bennett J , Johnstone R , Byrne P , et al. The effects of antiretroviral therapy on HIV‐1 RNA loads in seminal plasma in HIV‐positive patients with and without urethritis. AIDS. 2002;16(2):219–25. 11807306 10.1097/00002030-200201250-00011

[87] Chen JS , Matoga M , Massa C , Ndalama B , Jere E , Tegha G , et al. Back to the future: even in the ART era, men co‐infected with HIV and urethritis pose a potential transmission threat. HIV Research for Prevention (HIVR4P); October 21‐25, 2018; Madrid, Spain.

[88] Matoga M , Chen JS , Massa C , Ndalama B , Jere E , Tegha G , et al. Test and Treat in Malawi: HIV Seminal Viral Load Response to ART Initiation Among Men Co‐infected with Urethritis. HIV Research for Prevention (HIVR4P); October 21‐25, 2018; Madrid, Spain.

[89] Kelley CF , Haaland RE , Patel P , Evans‐Strickfaden T , Farshy C , Hanson D , et al. HIV‐1 RNA rectal shedding is reduced in men with low plasma HIV‐1 RNA viral loads and is not enhanced by sexually transmitted bacterial infections of the rectum. J Infect Dis. 2011;204(5):761–7. 21844302 10.1093/infdis/jir400 PMC3156109

[90] Davies O , Costelloe S , Cross G , Dew T , O'Shea S , White J , et al. Impact of rectal gonorrhoea and chlamydia on HIV viral load in the rectum: potential significance for onward transmission. Int J STD AIDS. 2017;28(10):1034–7. 28081680 10.1177/0956462416686513

[91] Ho YC , Shan L , Hosmane NN , Wang J , Laskey SB , Rosenbloom DI , et al. Replication‐competent noninduced proviruses in the latent reservoir increase barrier to HIV‐1 cure. Cell. 2013;155(3):540–51. 24243014 10.1016/j.cell.2013.09.020 PMC3896327

[92] Dyer JR , Eron JJ , Hoffman IF , Kazembe P , Vernazza PL , Nkata E , et al. Association of CD4 cell depletion and elevated blood and seminal plasma human immunodeficiency virus type 1 (HIV‐1) RNA concentrations with genital ulcer disease in HIV‐1‐infected men in Malawi. J Infect Dis. 1998;177(1):224–7. 9419194 10.1086/517359

[93] Buchacz K , Patel P , Taylor M , Kerndt PR , Byers RH , Holmberg SD , et al. Syphilis increases HIV viral load and decreases CD4 cell counts in HIV‐infected patients with new syphilis infections. AIDS. 2004;18(15):2075–9. 15577629 10.1097/00002030-200410210-00012 PMC6763620

[94] Lingappa JR , Baeten JM , Wald A , Hughes JP , Thomas KK , Mujugira A , et al. Daily acyclovir for HIV‐1 disease progression in people dually infected with HIV‐1 and herpes simplex virus type 2: a randomised placebo‐controlled trial. Lancet. 2010;375(9717):824–33. 20153888 10.1016/S0140-6736(09)62038-9 PMC2877592

[95] Champredon D , Bellan SE , Delva W , Hunt S , Shi CF , Smieja M , et al. The effect of sexually transmitted co‐infections on HIV viral load amongst individuals on antiretroviral therapy: a systematic review and meta‐analysis. BMC Infect Dis. 2015;15:249. 26123030 10.1186/s12879-015-0961-5 PMC4486691

[96] Grant RM , Lama JR , Anderson PL , McMahan V , Liu AY , Vargas L , et al. Preexposure chemoprophylaxis for HIV prevention in men who have sex with men. N Engl J Med. 2010;363(27):2587–99. 21091279 10.1056/NEJMoa1011205 PMC3079639

[97] McCormack S , Dunn DT , Desai M , Dolling DI , Gafos M , Gilson R , et al. Pre‐exposure prophylaxis to prevent the acquisition of HIV‐1 infection (PROUD): effectiveness results from the pilot phase of a pragmatic open‐label randomised trial. Lancet. 2016;387(10013):53–60. 26364263 10.1016/S0140-6736(15)00056-2 PMC4700047

[98] Molina JM , Charreau I , Spire B , Cotte L , Chas J , Capitant C , et al. Efficacy, safety, and effect on sexual behaviour of on‐demand pre‐exposure prophylaxis for HIV in men who have sex with men: an observational cohort study. Lancet HIV. 2017;4(9):e402–10. 28747274 10.1016/S2352-3018(17)30089-9

[99] Janes H , Corey L , Ramjee G , Carpp LN , Lombard C , Cohen MS , et al. Weighing the evidence of efficacy of oral PrEP for HIV prevention in women in Southern Africa. AIDS Res Hum Retroviruses. 2018;34(8):645–56. 29732896 10.1089/aid.2018.0031 PMC6080090

[100] Center for Disease Control and Prevention . Preexposure Prophylaxis for the Prevention of HIV Infection in the U.S.: 2017 Clinical Practice Guideline. Department of Health and Human Services. Epub Published online March 2018.

[101] Traeger MW , Schroeder SE , Wright EJ , Hellard ME , Cornelisse VJ , Doyle JS , et al. Effects of pre‐exposure prophylaxis for the prevention of human immunodeficiency virus infection on sexual risk behaviour in men who have sex with men: a systematic review and meta‐analysis. Clin Infect Dis. 2018;67(5):676–86. 29509889 10.1093/cid/ciy182

[102] Kojima N , Davey DJ , Klausner JD . Pre‐exposure prophylaxis for HIV infection and new sexually transmitted infections among men who have sex with men. AIDS. 2016;30(14):2251–2. 27314179 10.1097/QAD.0000000000001185

[103] Harawa NT , Holloway IW , Leibowitz A , Weiss R , Gildner J , Landovitz RJ , et al. Serious concerns regarding a meta‐analysis of preexposure prophylaxis use and STI acquisition. AIDS. 2017;31(5):739–40. 28225452 10.1097/QAD.0000000000001386 PMC5580999

[104] Traeger MW , Cornelisse VJ , Asselin J , Price B , Roth NJ , Willcox J , et al. Association of HIV preexposure prophylaxis with incidence of sexually transmitted infections among individuals at high risk of HIV infection. JAMA. 2019;321(14):1380–90. 30964528 10.1001/jama.2019.2947 PMC6459111

[105] Chow EPF , Cornelisse VJ , Williamson DA , Priest D , Hocking JS , Bradshaw CS , et al. Kissing may be an important and neglected risk factor for oropharyngeal gonorrhoea: a cross‐sectional study in men who have sex with men. Sex Transm Infect. 2019. 10.1136/sextrans-2018-053896 31073095

[106] Volk JE , Marcus JL , Phengrasamy T , Blechinger D , Nguyen DP , Follansbee S , et al. No new HIV infections with increasing use of HIV preexposure prophylaxis in a clinical practice setting. Clin Infect Dis. 2015;61(10):1601–3. 26334052 10.1093/cid/civ778 PMC4809999

[107] Baeten JM , Donnell D , Ndase P , Mugo NR , Campbell JD , Wangisi J , et al. Antiretroviral prophylaxis for HIV prevention in heterosexual men and women. N Engl J Med. 2012;367(5):399–410. 22784037 10.1056/NEJMoa1108524 PMC3770474

[108] Abdool Karim Q , Abdool Karim SS , Frohlich JA , Grobler AC , Baxter C , Mansoor LE , et al. Effectiveness and safety of tenofovir gel, an antiretroviral microbicide, for the prevention of HIV infection in women. Science. 2010;329(5996):1168–74. 20643915 10.1126/science.1193748 PMC3001187

[109] Baeten JM , Palanee‐Phillips T , Brown ER , Schwartz K , Soto‐Torres LE , Govender V , et al. Use of a vaginal ring containing dapivirine for HIV‐1 prevention in women. N Engl J Med. 2016;375(22):2121–32. 26900902 10.1056/NEJMoa1506110 PMC4993693

[110] Marrazzo JM , Ramjee G , Richardson BA , Gomez K , Mgodi N , Nair G , et al. Tenofovir‐based preexposure prophylaxis for HIV infection among African women. N Engl J Med. 2015;372(6):509–18. 25651245 10.1056/NEJMoa1402269 PMC4341965

[111] Thigpen MC , Kebaabetswe PM , Paxton LA , Smith DK , Rose CE , Segolodi TM , et al. Antiretroviral preexposure prophylaxis for heterosexual HIV transmission in Botswana. N Engl J Med. 2012;367(5):423–34. 22784038 10.1056/NEJMoa1110711

[112] Van Damme L , Corneli A , Ahmed K , Agot K , Lombaard J , Kapiga S , et al. Preexposure prophylaxis for HIV infection among African women. N Engl J Med. 2012;367(5):411–22. 22784040 10.1056/NEJMoa1202614 PMC3687217

[113] van der Straten A , Van Damme L , Haberer JE , Bangsberg DR . Unraveling the divergent results of pre‐exposure prophylaxis trials for HIV prevention. AIDS. 2012;26(7):F13–9. 22333749 10.1097/QAD.0b013e3283522272

[114] Nel A , van Niekerk N , Kapiga S , Bekker LG , Gama C , Gill K , et al. Safety and efficacy of a dapivirine vaginal ring for HIV prevention in women. N Engl J Med. 2016;375(22):2133–43. 27959766 10.1056/NEJMoa1602046

[115] Hare CB , Coll J , Ruane P , Molina JM , Mayer KH , Jessen H , et al. Discover trial: TAF noninferior to TDF for HIV PrEP. Conference on Retroviruses and Opportunistic Infections (CROI); Seattle, WA. March 4‐7, 2019.

[116] Landovitz RJ , Li S , Grinsztejn B , Dawood H , Liu AY , Magnus M , et al. Safety, tolerability, and pharmacokinetics of long‐acting injectable cabotegravir in low‐risk HIV‐uninfected individuals: HPTN 077, a phase 2a randomized controlled trial. PLoS Med. 2018;15(11):e1002690. 30408115 10.1371/journal.pmed.1002690 PMC6224042

[117] Barrett SE , Teller RS , Forster SP , Li L , Mackey MA , Skomski D , et al. Extended‐duration MK‐8591‐eluting implant as a candidate for hiv treatment and prevention. Antimicrob Agents Chemother. 2018;62(10):e01058–18; DOI: 10.1128/AAC.01058-18 30012772 PMC6153840

[118] Li W , Terry RN , Tang J , Feng MR , Schwendeman SP , Prausnitz MR . Rapidly separable microneedle patch for the sustained release of a contraceptive. Nat Biomed Eng. 2019;3(3):220–9. 30948808 10.1038/s41551-018-0337-4

[119] Anderson SJ , Cherutich P , Kilonzo N , Cremin I , Fecht D , Kimanga D , et al. Maximising the effect of combination HIV prevention through prioritisation of the people and places in greatest need: a modelling study. Lancet. 2014;384(9939):249–56. 25042235 10.1016/S0140-6736(14)61053-9

[120] Okano JT , Robbins D , Palk L , Gerstoft J , Obel N , Blower S . Testing the hypothesis that treatment can eliminate HIV: a nationwide, population‐based study of the Danish HIV epidemic in men who have sex with men. Lancet Infect Dis. 2016;16(7):789–96. 27174504 10.1016/S1473-3099(16)30022-6 PMC5242518

[121] Abuelezam NN , McCormick AW , Fussell T , Afriyie AN , Wood R , DeGruttola V , et al. Can the heterosexual HIV epidemic be eliminated in South Africa using combination prevention? A Modelling Analysis Am J Epidemiol. 2016;184(3):239–48. 27416841 10.1093/aje/kwv344 PMC4967594

[122] Chesson HW , Kidd S , Bernstein KT , Fanfair RN , Gift TL . The cost‐effectiveness of syphilis screening among men who have sex with men: an exploratory modelling analysis. Sex Transm Dis. 2016;43(7):429–32. 27322043 10.1097/OLQ.0000000000000461 PMC6745685

[123] Jenness SM , Weiss KM , Goodreau SM , Gift T , Chesson H , Hoover KW , et al. Incidence of gonorrhea and chlamydia following human immunodeficiency virus preexposure prophylaxis among men who have sex with men: a modelling study. Clin Infect Dis. 2017;65(5):712–8. 28505240 10.1093/cid/cix439 PMC5848234

[124] Bolan RK , Beymer MR , Weiss RE , Flynn RP , Leibowitz AA , Klausner JD . Doxycycline prophylaxis to reduce incident syphilis among HIV‐infected men who have sex with men who continue to engage in high‐risk sex: a randomized, controlled pilot study. Sex Transm Dis. 2015;42(2):98–103. 25585069 10.1097/OLQ.0000000000000216 PMC4295649

[125] Molina JM , Charreau I , Chidiac C , Pialoux G , Cua E , Delaugerre C , et al. Post‐exposure prophylaxis with doxycycline to prevent sexually transmitted infections in men who have sex with men: an open‐label randomised substudy of the ANRS IPERGAY trial. Lancet Infect Dis. 2018;18(3):308–17. 29229440 10.1016/S1473-3099(17)30725-9

[126] Archin NM , Liberty AL , Kashuba AD , Choudhary SK , Kuruc JD , Crooks AM , et al. Administration of vorinostat disrupts HIV‐1 latency in patients on antiretroviral therapy. Nature. 2012;487(7408):482–5. 22837004 10.1038/nature11286 PMC3704185

[127] Margolis DM , Garcia JV , Hazuda DJ , Haynes BF . Latency reversal and viral clearance to cure HIV‐1. Science. 2016;353(6297): aaf6517. 27463679 10.1126/science.aaf6517 PMC5021637

[128] Cohen MS , Corey L . Broadly neutralizing antibodies to prevent HIV‐1. Science. 2017;358(6359):46–7. 28983040 10.1126/science.aap8131

[129] Smith DK , Chang MH , Duffus WA , Okoye S , Weissman S . Missed opportunities to prescribe preexposure prophylaxis in South Carolina, 2013–2016. Clin Infect Dis. 2019;68(1):37–42. 29790923 10.1093/cid/ciy441

